# Playful Minds Under Pressure? Exploring Links Between Playfulness, Stress, and Satisfaction in Employees and Students

**DOI:** 10.3390/healthcare14040431

**Published:** 2026-02-09

**Authors:** Rebekka Sendatzki, Kay Brauer, René T. Proyer

**Affiliations:** Department of Psychology, Martin Luther University Halle-Wittenberg, 06108 Halle (Saale), Germany; kay.brauer@psych.uni-halle.de (K.B.); rene.proyer@psych.uni-halle.de (R.T.P.)

**Keywords:** playfulness, job satisfaction, stress, well-being, employees, healthcare, students

## Abstract

**Background/Objectives:** University students and working professionals, especially those preparing for or employed in healthcare, face substantial psychological demands. Identifying low-cost, easily deployable personal resources to buffer stress and enhance satisfaction is therefore a priority for positive-psychology research and practice. Adult playfulness, the disposition to (re)frame everyday situations in an entertaining, interesting, or meaningful way, may represent such a resource, but evidence from applied settings remains scarce. We investigated how four facets of playfulness relate to perceived stress and job/academic satisfaction across study and work contexts, as well as whether these associations differ by healthcare affiliation. **Methods:** We analyzed two samples comprising 499 employed adults from diverse occupational backgrounds and 635 university students (*N*_total_ = 1134). Participants completed measures of playfulness, perceived stress, and job/academic satisfaction. **Results:** Multi-group confirmatory factor analyses supported scalar measurement invariance across occupational status and healthcare affiliation. Structural equation models indicated that lighthearted playfulness was associated with lower perceived stress. Associations between playfulness facets and satisfaction were weak overall and were primarily observed among working adults. We found no evidence for moderation by healthcare affiliation. **Conclusions:** Given that playfulness is a relatively stable disposition whereas stress and satisfaction. These findings underscore the importance of a differentiated conceptualization of playfulness and point to the potential value for future research examining facet-specific, context-sensitive applications in education and practice.

## 1. Introduction

The modern workplace and educational environments have become increasingly demanding, which places significant pressures on employees and students alike [1,2]. This heightened stress negatively affects well-being, productivity, and overall life satisfaction (e.g., [1,3]). Stress-related issues not only impair individual health but also impose substantial economic burdens globally, which highlights the need to identify protective factors that foster resilience and well-being in occupational and academic contexts (e.g., [2]).

Several established frameworks emphasize the role of personal resources in shaping how individuals experience and cope with stress; these include the transactional stress model [4], the job demands–resources model [5], and positive psychology approaches such as flow theory and the broaden-and-build theory (e.g., [6,7]). Across these perspectives, individual differences in appraisal, coping tendencies, and emotion-related processes are considered relevant for understanding stress, as they shape how demands are interpreted and regulated rather than reflecting the objective presence of stressors alone.

Anchored in positive psychology, we view adult playfulness as a potential resource that can broaden positive emotion and support adaptive coping in study and work contexts (e.g., [7,8,9]. Proyer suggests that playfulness is an individual characteristic that involves (re)framing situations such that they are perceived not as essentially stressful, but as entertaining, intellectually stimulating, and/or personally interesting [10]. From this perspective, playfulness can be situated within broader models of stress and well-being. For example, by influencing how situational demands are interpreted and engaged with, rather than by reducing demands themselves.

This study integrates insights from organizational, positive, and personality psychology to examine relationships between playfulness, perceived stress, and satisfaction among two populations: working professionals and university students. We further explored whether these relationships vary by affiliation. Healthcare contexts are characterized by high psychological demands, including shift work, responsibility for others’ well-being, emotional labor), rather than primarily task-based or time limited [11,12,13,14]. In such environments, playfulness may be particularly relevant in such environments, where cognitive flexibility and reinterpreting situations are appraised, regulated, and socially negotiated.

Although playfulness is not a standalone answer to stress or (dis)satisfaction at work or university, this study aims to provide initial insights of where and for whom it may matter. Such preliminary evidence can inform low-threshold activities that support well-being in educational and occupational settings, ultimately guiding future research and practical applications.

### 1.1. Adult Playfulness

Historically, research on playfulness has focused mainly on children. In recent decades, however, increasing attention has been paid to its expression and correlates in adulthood. Defining adult playfulness requires capturing its essence while distinguishing it from related constructs such as, for example, humor, curiosity, or creativity [15]. Proyer [10] defines playfulness as “an individual differences variable that allows people to frame or reframe everyday situations in a way such that they experience them as entertaining, and/or intellectually stimulating, and/or personally interesting” (p. 114). This emphasizes individuals’ capacity to interpret experiences playfully and derive amusement, intellectual stimulation, and personal interest from daily situations.

To describe this multidimensional construct, Proyer [10] proposed the OLIW model of adult playfulness, comprising four facets: *other-directed* (playfulness expressed in social interactions that ease tension and foster joy), *lighthearted* (a spontaneous, carefree attitude favoring improvisation over rigid planning), *intellectual* (enjoyment in engaging with new ideas and creative problem-solving), and *whimsical* (a preference for the unconventional and extraordinary). The OLIW model provides a framework to study how facets of playfulness relate to psychological and behavioral outcomes. Prior studies have linked playfulness to higher well-being, health, interpersonal functioning, creativity and innovation [16,17,18,19,20,21].

Playfulness is increasingly recognized as a personal resource within organizational and educational contexts. Empirical findings suggest associations with motivational, interpersonal, and coping-related outcomes in both work and university settings [22,23,24,25]. Although playfulness relates to indicators of positive psychological functioning, some studies associate it with counterproductive behaviors such as procrastination [26], and findings regarding job performance have been mixed [27,28]. Increasingly, such inconsistencies are attributed to meaningful differences between the different facets of playfulness. In particular, other-directed, lighthearted, and intellectual playfulness facets tend to show more consistent links to adaptive coping and psychological functioning, whereas whimsical playfulness appears comparatively less consistently related to positive outcomes (e.g., [9,29]).

These findings underline that playfulness is a multifaceted construct whose expression and correlates vary across contexts such as work and study. Understanding how these facets relate to key indicators of functioning, including perceived stress and satisfaction with one’s occupation, therefore represents an important next step.

### 1.2. Playfulness in Relation to Stress and Satisfaction

#### 1.2.1. Playfulness and Stress

Lazarus and Folkman [4] define psychological stress as “a relationship between the person and the environment that is appraised by the person as taxing or exceeding his or her resources and endangering his or her well-being” (p. 21). This definition highlights two key characteristics: (1) stress arises from person–environment interactions, which means that individuals differ in how they perceive and experience identical stressors; and (2) personal resources significantly influence how threatening stress will be perceived to be. Given this emphasis on individual appraisal, it could be argued that playfulness relates to dealing with and experiencing stress. Playfulness, defined as the tendency to frame or reframe experiences in a more positive or engaging manner, could act as a personal resource that shapes stress appraisal rather than eliminating the stressors themselves [30].

According to the broaden-and-build theory [7], positive emotions broaden thought–action repertoires and help build psychological and social resources. Fredrickson [7] argues that playfulness can promote more flexible appraisals by fostering positive emotions and allowing individuals to explore alternative interpretations of challenging situations. Similarly, the concept of flow [6] suggests that playfulness facilitates engagement and intrinsic motivation, thus supporting deeper involvement in activities that may mitigate strain.

Empirical studies support these theoretical accounts. For instance, Magnuson and Barnett [31] found that playful university students reported lower stress and used more active coping strategies than their less playful peers. Similarly, Chang et al. [32] reported negative correlations between playfulness and perceived stress (*r* = −0.23) among university students, which indicates that playful students may appraise stressful situations as less threatening. More recently, Clifford et al. [33] found that more playful adults reported higher self-efficacy, lower helplessness, and more adaptive coping during the COVID-19 pandemic. Evidence from workplace settings also links playfulness to more adaptive coping [9], which reinforces its role as a personal resource in managing everyday demands. Across studies, correlations between playfulness and stress typically show correlation effect sizes between 0.20 and 0.35. However, little is known about which facets of playfulness contribute most strongly to this association.

#### 1.2.2. Playfulness and Job/Academic Satisfaction

Domain-specific satisfaction, such as job or academic satisfaction, is a central component of well-being within positive psychology. Playfulness may relate to higher satisfaction by fostering engagement and intrinsic motivation [6] and by functioning as a personal resource within the job demands–resources model [5]. Empirical evidence links playfulness to life satisfaction [9,34,35], happiness and well-being [17,34,36,37], and mental health [19] across age groups. Experimental studies further demonstrate that short playfulness interventions (i.e., 7-day self-directed activities) can enhance well-being and reduce depressiveness [21]. Notably, however, whimsical playfulness shows numerically weaker or inconsistent links to positive outcomes [9,17,19,34,36], which indicates heterogeneity across facets and the importance of considering the facet approach to playfulness in adults.

In occupational contexts, evidence on playfulness and satisfaction is limited. Yu et al. [28] reported a moderate correlation between global playfulness and job satisfaction (*r* = 0.35) in a large Taiwanese sample (*N* = 1493) and early work on “play at work” showed similar patterns [38] (*r* = 0.22). More recent research on related constructs, support these associations. Employees who engage in playful work design—that is, proactively shaping their work to increase enjoyment or challenge—report higher work engagement and initiative, and they are perceived by colleagues as more engaged and creative [26,39]. Although job satisfaction itself was not assessed, such evidence suggests that a playful orientation at work may support more positive work experiences.

Overall, prior findings indicate small-to-moderate positive associations between playfulness and satisfaction-related outcomes. Yet most studies rely on global measures, thus limiting insight into which playfulness facets matter most in academic and occupational settings.

### 1.3. Present Study

#### 1.3.1. Rationale and Research Questions

In the present study, we examined how the four OLIW facets of playfulness relate to perceived stress and two domains of satisfaction (general and growth satisfaction). We collected data from working professionals and university students to compare them and examine whether occupational status plays a role for the findings. Prior studies have rarely addressed these relationships at the facet level or compared results across different populations, despite robust evidence that playfulness plays a role in both academic and occupational domains [22,24,25,35,40,41].

We selected students and professionals as target groups because they share important parallels, including comparable workloads, the need for specific skills to complete tasks, and outcome-dependent rewards such as grades or salary [42]. At the same time, these groups differ in the degree of structure and accountability inherent to their roles. Comparing them therefore allows for a broader understanding of how playfulness functions across similar but distinct environments.

We further explored whether an affiliation with a healthcare profession or course of education moderated the results. Healthcare settings are characterized by high psychological and emotional demands, including shift work and high workloads, responsibility for others’ well-being, and emotionally intense interactions with patients and their families [11,12,13]. Stress in healthcare is therefore often interpersonal, emotionally taxing, and only partly controllable, rather than primarily task-based or time-limited as in other high-stress jobs (e.g., [14]). In such contexts, personal resources such as playfulness may be particularly relevant not by reducing demands per se, but by shaping how emotionally demanding situations are experienced and navigated—for example, through processes related to cognitive reappraisal, emotion regulation, and social connectedness.

Prior research has shown that healthcare professionals frequently use humor to cope with stressors, regulate difficult emotions, and maintain supportive relationships within their teams [43,44,45,46]. Reviews and meta-analytic findings link positive humor use to lower stress and burnout as well as higher coping effectiveness, workgroup cohesion, and job satisfaction [44,47]. Although humor and playfulness are distinct constructs, playfulness may provide the flexible mindset that facilitates humorous reframing [48]. Taken together, this study builds on playfulness in healthcare, we examined the moderating role of healthcare affiliation in an exploratory manner.

#### 1.3.2. Expectations

Drawing on theoretical perspectives such as the broaden-and-build theory [7], flow theory [6], and the job demands–resources model [5]), as well as prior empirical work (e.g., [17,19,28]), we expected positive associations between playfulness and satisfaction.

Other-directed playfulness involves engaging playfully with others to ease tension and foster positive interactions. Because this facet emphasizes social connection, it may buffer stress by reducing interpersonal tension and enhancing social support in demanding situations [16]. We therefore expected this facet to relate to lower stress and greater satisfaction, as it might act as interpersonal resource in both academic and occupational contexts.

Lighthearted playfulness reflects a carefree, spontaneous attitude, should relate to lower perceived stress and greater satisfaction, as such individuals tend to view challenges as more manageable (e.g., [9]). Lighthearted playfulness may also be indirectly related to satisfaction insofar as reduced strain and a less burdened appraisal of daily demands can support more positive evaluations of one’s work or study experience. Accordingly, we expected lighthearted playfulness to be negatively associated with perceived stress and positively associated with satisfaction.

Intellectual playfulness involves a preference for engaging with new ideas, problem-solving, and cognitive challenges. We hypothesized that this facet is positively related to satisfaction, as intellectually playful individuals find fulfillment in challenging tasks and learning opportunities (e.g., [41]). This may be particularly relevant for growth-related satisfaction and, among students, for academic satisfaction, where engagement with cognitively demanding material is central.

Whimsical playfulness, characterized by a preference for the unconventional and extraordinary, may be less compatible with structured academic and workplace environments (e.g., [25]) and therefore show weaker or context-dependent associations with satisfaction. Consistent with previous findings (e.g., [9,17,34]), we expected weak positive or even negative relationships with satisfaction and potentially higher stress, thus reflecting a possible mismatch between unconventional self-expression and norm-bound contexts.

Taken together, we expected that lighthearted playfulness, and to a lesser extent other-directed and intellectual playfulness, would be linked to lower stress and higher satisfaction with effect sizes in the small-to-moderate range. In contrast, we examined the relations with whimsical playfulness exploratorily, given its inconsistent associations with positive outcomes in prior research. We also conducted analyses involving occupational status and healthcare affiliation with an explicit exploratory focus, given the limited prior evidence on facet-specific playfulness in these contexts.

## 2. Method

### 2.1. Participants

We collected data from students and professionals in Germany. Sample 1 comprised 635 university students (80.5% women, 18.0% men, 1.6% non-binary) and Sample 2 consisted of 499 professionals (69.3% women, 29.7% men, 1.0% non-binary). Detailed descriptions for the samples (age, education, working hours, and study/work area) are provided in Table 1. In short, the professionals were on average in their late 30s and the students in their early 20s. Of the students, 82.2% were pursuing a bachelor’s degree, while the remaining 17.8% were working towards a master’s degree. Among the professionals, 68.9% were solely working, 1.6% were in vocational training, and 29.5% were students with more than 20 working hours per week. Nearly two thirds of the professionals were working at least 35 h per week (*M* = 35.7 h/week), and they had 2.9 h of overtime per week on average. The sample sizes meet the recommendations for estimating stable correlations between manifest and latent variables [49,50].

### 2.2. Instruments

#### 2.2.1. Playfulness

The 28-item OLIW questionnaire [10] assesses four facets of playfulness: *Other-directed* (e.g., “I can use my playfulness to do something nice for other people, or to cheer them up”), *Lighthearted* (e.g., “Many people take their lives too seriously; when things don’t work you just have to improvise”), *Intellectual* (e.g., “If I want to develop a new idea further and think about it, I like to do this in a playful manner”), and *Whimsical* (e.g., “I have the reputation of being somewhat unusual or flamboyant”) with 7 items each. Each item is rated on a 7-point scale (1 = *strongly disagree*, 7 = *strongly agree*). The questionnaire demonstrates satisfactory reliability (internal consistency, test–retest correlations of ≥0.74 and ≥0.67 for 1- and 3-month intervals, and inter-rater agreement), factorial validity, convergence with self- and peer reports, daily behavior ratings, and other playfulness measures (e.g., [10,51]). The OLIW questionnaire is frequently used in playfulness research [17,36]. Internal consistencies for all instruments obtained in the present samples are reported in Table 2.

#### 2.2.2. Perceived Stress

We assessed perceived stress with the *Perceived Occupational Stress Scale* (POS; [52]), which includes four items that assess the subjective experience of stress at work (e.g., “My work is stressful”). Participants give their responses on a 5-point Likert scale (1 = *strongly disagree*, 5 = *strongly agree*). The POS has demonstrated good internal consistency (α = 0.82), strong test–retest reliability (*r* = 0.86 for four weeks), and convergent validity [52]. For students, we adapted the item wording to the academic context (e.g., “My studies are stressful”). As there is no validated German version of the POS, we translated the items using a standard back-translation approach. For an initial validation, a confirmatory factor analysis (CFA; WLSMV estimator) supported the expected unidimensional structure of the POS in the total sample (CFI = 0.998, TLI = 0.995, RMSEA = 0.065, SRMR = 0.023), as well as in the student and professional subsamples (CFI = 0.998/0.996, TLI = 0.996/0.989, RMSEA = 0.054/0.095, SRMR = 0.022/0.032). All standardized factor loadings were high, ranging from 0.69 to 0.89.

#### 2.2.3. Job and Academic Satisfaction

We used a shortened version of the *Job Diagnostic Survey* (JDS, [53]) to assess job satisfaction (employees) and academic satisfaction (students). We used four items of the JDS to assess *general job satisfaction* (2 items, “satisfied with job”) and *growth satisfaction* (2 items, “satisfied with development opportunities”). Participants gave their answers on a 6-point rating scale (1 = *strongly disagree*, 6 = *strongly agree*). For students, we changed the item wordings slightly to accommodate their situation (e.g., “I am very satisfied with my studies”). Hackman and Oldham [53] reported acceptable internal consistencies with α = 0.76 for job satisfaction and 0.84 for growth. For the original JDS, Hackman and Oldham reported overlap with supervisor (*r*_Mdn_ = 0.51) and observer ratings (*r*_Mdn_ = 0.63), and convergent and discriminant validity [53].

#### 2.2.4. Healthcare Affiliation

We dummy-coded healthcare affiliation (1 = affiliation, 0 = none/low). The “affiliated” category comprised all respondents who studied or worked in medicine, nursing, pharmacy, clinical or health psychology, physiotherapy, occupational therapy, or other roles involving direct patient care or clinical services (*n* = 154: 96 professionals and 58 students), whereas every other study major or occupation was coded 0 (*n* = 980, 403 professionals and 577 students).

### 2.3. Procedure

We advertised the study on-campus, through social media, via leaflets, and on the authors’ department website. Participation was voluntary, and all participants provided informed consent before taking part. No financial compensation was provided; however, psychology students could earn course credit. To be eligible, participants needed to be 18 years or older, and either working at least 20 h per week (professionals) or studying at university (students). Data collection took place in 2023. We conducted the study in accordance with the ethical standards of the German Psychological Society (DGPs) and the Declaration of Helsinki. Ethical approval was not required according to institutional guidelines and local legislation.

### 2.4. Data Analysis

We first tested the measurement model of the seven latent constructs (four OLIW facets, stress, and general and growth satisfaction) by conducting a CFA for students and professionals separately. We used the WLSMV estimator to account for the ordinal response formats of our scales [54]. Model fit was evaluated using standard fit indices (CFI, TLI, RMSEA, and SRMR). As no established cutoffs exist for evaluating CFA models estimated with WLSMV, we interpreted model fit according to Hu and Bentler’s [55] guidelines, while noting that these thresholds are typically not met with WLSMV because it yields more accurate but less favorable fit indices [56].

Second, we examined measurement invariance across (a) students versus professionals and (b) those with versus without healthcare affiliation. We examined (I) configural invariance (i.e., invariance regarding the number of factors); (II) metric invariance (i.e., invariant item–factor loadings); and finally (III) scalar invariance (i.e., invariance of item intercepts and latent means). We again used the seven latent constructs and the WLSMV estimator. In line with Chen’s [57] recommendations, we rejected metric invariance when ΔCFI ≥ 0.010 and ΔRMSEA ≥ 0.015 or ΔSRMR ≥ 0.030 and rejected scalar invariance when ΔCFI ≥ 0.010 and ΔRMSEA ≥ 0.015 or ΔSRMR ≥ 0.010.

Finally, we estimated structural equation models (SEMs) to examine the relationships between playfulness, stress, and satisfaction. Although no directionality can be estimated using cross-sectional data, we modelled the OLIW facets as predictors given their relative temporal stability compared to stress and satisfaction. For the SEMs, we used the maximum likelihood robust (MLR) estimator because it is robust to non-normality, suitable for complex models, and more appropriate for testing associations between latent variables [58]. We tested two models: an unconstrained model allowing parameters to vary freely between students and professionals, and a constrained model in which the regression coefficients were set to be equal across groups (i.e., assuming equal associations between playfulness and outcome variables in both groups). We compared the models using Satorra–Bentler-corrected χ^2^ difference tests to determine whether the path coefficients differed between groups; a significant difference test indicates that the groups cannot be considered equivalent. We used the same procedure for the moderating role of healthcare affiliation. For all analyses, we used Mplus version 8.8 [58]. Data and analysis code are available on the OSF website (https://osf.io/fa2zb, accessed on 22 December 2025).

## 3. Results

### 3.1. Measurement Model of the Seven Latent Constructs

Our CFAs showed moderate fit for students, with χ^2^(*df* = 630) = 13,920.25, CFI = 0.887, TLI = 0.876, RMSEA = 0.064 (90% CI [0.061; 0.067]), and SRMR = 0.060. Similarly, we found moderate fit for professionals, with χ^2^(*df* = 573) = 1763.89, CFI = 0.883, TLI = 0.872, RMSEA = 0.065 (90% CI [0.061; 0.068]), and SRMR = 0.064. As expected with WLSMV estimation, CFI and TLI were slightly below conventional cutoffs (≥0.90), whereas RMSEA and SRMR values were within the acceptable range for other estimators. Given the model’s theoretical foundation, its considerable complexity (seven latent constructs, 36 items), and consistent performance across multiple indices, we considered the measurement model to be sufficient for invariance and structural analyses.

### 3.2. Descriptive Statistics

Internal consistency (McDonald’s ω) ranged from 0.72 to 0.84, except for intellectual playfulness (ω = 0.62), which is consistent with prior studies (e.g., [25,29]). Score distributions of our variables were comparable to prior studies, although stress levels were higher than in the original normative sample (*d* = 0.58; [52]). Initial correlational analyses of the total sample showed that higher stress was associated with lower lighthearted playfulness (*r* = −0.19, *p* < 0.001) and lower job/academic and growth satisfaction (*r* = −0.29 and −0.14, *p*s < 0.001). All descriptive statistics are shown in Table 2.

### 3.3. Measurement Invariance

Measurement invariance analyses across (a) students and professionals and (b) those with and without healthcare affiliation indicated scalar invariance for both occupational groups (ΔCFI ≤ 0.011, ΔRMSEA ≤ 0.002, and ΔSRMR ≤ 0.001) and healthcare affiliation (ΔCFI ≤ 0.050, ΔRMSEA ≤ 0.001, and ΔSRMR ≤ 0.002). All coefficients are shown in Table 3. Mean comparisons showed that students reported higher other-directed playfulness (*d* = 0.32) and stress (*d* = 0.35), whereas professionals were more satisfied with their job than students were with their studies (*d* = 0.28). There were no differences in our variables for those with and without healthcare affiliation, except that healthcare workers/students reported having slightly higher job/academic satisfaction (*d* = 0.23, *p* = 0.008).

### 3.4. Relations of Playfulness with Stress and Satisfaction

We first tested whether the SEM was invariant across students and professionals. Constraining all paths to be equal across students and professionals significantly worsened the fit (Δχ^2^[*df* = 12] = 22.82, *p* = 0.029), thus demonstrating group differences. In both groups, lighthearted playfulness was related to lower stress (β_students/professionals_ = −0.40/−0.28, *p*s < 0.001). In contrast, whimsical playfulness showed a small positive association with stress that was comparable in both groups (β = 0.15), reaching statistical significance only in students (*p*_students/professionals_ = 0.035/0.082). See Table 4 for all results.

For satisfaction, statistically significant associations emerged only for the professionals. Other-directed playfulness was positively related to both job and growth satisfaction (β_professionals_ = 0.36 and 0.32, *p*s ≤ 0.008). Among students, associations with satisfaction were negligible (|β_students_| ≤ 0.12, *p*s ≥ 0.162).

### 3.5. Healthcare Affiliation as Moderator

Constraining all paths to be equal across those with and without healthcare affiliation did not worsen the model fit (Δχ^2^[*df* = 12] = 15.54, *p* = 0.213). Hence, the associations between playfulness and stress and satisfaction did not depend on whether participants worked or studied in a healthcare-related field.

## 4. Discussion

Our study examined how the OLIW facets of playfulness relate to perceived stress and job or academic satisfaction among working professionals and university students. Overall, lighthearted playfulness was consistently associated with lower perceived stress across both students and professionals. Associations between playfulness facets and satisfaction were generally small and were primarily observed among working professionals, with other-directed playfulness showing positive links to job and growth satisfaction. Finally, healthcare affiliation did not moderate the associations between playfulness, stress, and satisfaction.

The measurement models showed acceptable fit, and scalar measurement invariance indicated that students and professionals interpreted the constructs similarly. Students showed slightly higher stress than professionals, and professionals showed higher satisfaction than students, but these differences were small in magnitude. This finding fits with the literature showing elevated perceived psychological burden and stress among students due to, among other reasons, academic pressure and transitional life circumstances (e.g., [59,60]).

### 4.1. Playfulness and Stress

The results showed a clear pattern regarding the associations between playfulness and stress. Lighthearted playfulness was linked to lower perceived stress, replicating earlier work linking playful attitudes to fewer stress symptoms and more adaptive coping strategies (e.g., [31,33]). Conceptually, lighthearted playfulness reflects a carefree and flexible approach to life, which may facilitate positive reappraisal and emotional regulation in stressful situations. According to the broaden-and-build theory [7], such positive emotions can expand coping repertoires and promote viewing stressors as manageable challenges rather than threats

In contrast, whimsical playfulness showed a small positive association with stress. Whimsical playfulness captures unconventionality and eccentricity [10], which may be in conflict with structured academic or workplace settings and thus create tension. This finding highlights that playfulness is not uniformly associated with positive outcomes, and that its contribution to managing stress and strain may partially depend on situational fit.

While the expected link between lighthearted playfulness and reduced stress was supported, other-directed and intellectual playfulness showed no meaningful associations with stress. Intellectual playfulness may be more relevant for cognitive outcomes such as creativity or problem-solving than for emotional outcomes like stress. The absence of robust associations for other-directed playfulness suggests that interpersonal types of playfulness may matter more for outcomes in the social domain than for stress appraisal. Future research could examine nonlinear patterns—particularly whether moderate levels of certain facets relate more favorably to stress than very low or very high levels.

### 4.2. Playfulness and Satisfaction

We only found one meaningful association for playfulness and satisfaction. Among professionals, other-directed playfulness was positively linked with both job and growth satisfaction, although it was unrelated to stress. This facet fosters positive interpersonal dynamics, social bonding, and satisfaction in close relationships [16], which may also translate to the workplace. Within the job demands–resources framework [5], other-directed playfulness may function as a social resource that enhances job satisfaction. These social exchanges may also create more opportunities for personal and professional growth, which is consistent with the observed link to growth satisfaction. The lack of comparable associations for other-directed playfulness with satisfaction among students could be attributed to differences in the social structure. Workplace teams offer stable team interactions that allow playful dynamics to accumulate, whereas university peer networks tend to be more transient.

### 4.3. Healthcare Affiliation

Our exploratory analyses yielded two notable findings regarding healthcare workers. First, healthcare affiliation did not moderate associations between playfulness and stress or satisfaction. This suggests that links between playfulness and processes such as cognitive appraisal, emotion regulation, or social connectedness may function similarly across occupational contexts, including those marked by high psychological and emotional demands [11,12,13].

Second, healthcare-affiliated participants did not report higher stress than others and showed slightly higher satisfaction. Given that overall stress in our sample was comparatively high relative to other POS-based studies (e.g., [52]), the absence of group differences likely reflects reduced contrast rather than unusually low strain in healthcare roles. Moreover, prior work has shown that substantial stress and high job satisfaction can co-occur in healthcare settings [12].

Several explanations may account for this pattern. Many healthcare roles involve strong job resources, such as team cohesion, supervisor and colleague support, and effective interprofessional collaboration, which can help maintain satisfaction despite high demands [11,61]. The healthy-worker effect provides another potential explanation, as individuals who remain in demanding clinical roles tend to have higher work ability and coping resources, while those with poorer well-being may leave the profession [62]. Self-selection may have further amplified this pattern, as highly stressed healthcare professionals are less likely to participate in a voluntary survey. Finally, our healthcare group was relatively small and heterogeneous, which limits the detection of more fine-grained differences.

### 4.4. Conceptual Considerations

Overall, our findings were less consistent than expected. Although methodological and sample-related factors may have contributed to the small effect sizes, the use of a differentiated measure of playfulness likely also played a role. Earlier studies often relied on global measures that blend playfulness with humor, positive affect, or creativity (e.g., [63,64]). Such conceptual overlap likely inflated previously reported associations with well-being and stress (see also, [15]).

Our findings therefore align with recent work demonstrating that playfulness is not uniformly linked to positive outcomes and that associations vary by facet and situational context [9,17,29,36,40]. In the present study, lighthearted playfulness showed the most consistent association with perceived stress, whereas other-directed playfulness was primarily related to satisfaction outcomes, underscoring that different facets may be relevant for distinct aspects of psychological functioning. At the same time, the comparatively small effect sizes should be interpreted in light of the fact that playfulness represents a relatively narrow, trait-like disposition, whereas perceived stress and satisfaction are strongly shaped by situational and structural factors. In addition, associations may not be strictly linear, particularly for facets such as lighthearted or whimsical playfulness, where both very low and very high expressions could be associated with strain depending on contextual fit.

The OLIW questionnaire also assesses playfulness as broad, domain-general dispositions rather than capturing context-specific expressions, such as playfulness at work or in academic settings. Contextualized measures, such as playful work design [26], may therefore show stronger and more consistent associations with well-being because they capture situationally relevant manifestations. Future work should therefore incorporate context-sensitive assessments to better understand how playful tendencies unfold across different environments.

Playfulness can also be stimulated by short interventions, which have been shown to reduce depressiveness and increase well-being [21]. These findings indicate that playfulness is not only a stable trait but also a malleable personal resource. Future studies could therefore build on the present correlational findings by experimentally enhancing specific facets, such as lighthearted or other-directed playfulness, to test whether increasing these tendencies reduces stress or increases satisfaction. Existing interventions have targeted playfulness in a broad sense (e.g., noting three playful things throughout the day); tailoring such activities to work contexts (e.g., noting playful experiences specifically at work) may further increase their utility in occupational settings.

### 4.5. Limitations and Future Directions

Key strengths of this study include its large and diverse samples, the comparison of two populations, and the use of a multidimensional measure of playfulness. Nevertheless, several limitations should be acknowledged.

First, the cross-sectional design precludes causal inference regarding whether playfulness influences stress and satisfaction or vice versa. Longitudinal or experience-sampling designs could capture within-person dynamics and clarify temporal mechanisms. Future research should also explore underlying processes and mechanisms, such as coping, positive emotion regulation, and social resource building.

In addition, the present analyses focused on the main effects of the playfulness facets. Although interactions between facets may be theoretically meaningful, testing such effects would require substantially more complex latent models and a large number of additional parameters, increasing the risk of false-positive findings. Given that the study was not designed or powered for this level of model complexity, facet-level interaction effects were not examined and remain an important avenue for future research.

Second, all variables were self-reported, which introduces common method bias. Although self-reports are well suited to capture subjective experiences such as perceived stress and satisfaction [65], future studies would benefit from incorporating additional data sources (e.g., peer reports or behavioral indicators).

Third, the student sample in particular showed a gender imbalance, which may limit the generalizability of the findings. However, gender was statistically controlled for in the analyses and our findings remained robust. This suggests that the observed associations are not driven by gender differences alone. Nevertheless, future studies should aim to replicate these findings in more gender-balanced samples to further strengthen generalizability and the robustness of our findings.

Fourth, our healthcare subsample was small and heterogeneous, which likely reduced statistical power for detecting moderation effects. Accordingly, the absence of moderation by healthcare affiliation should be interpreted with caution. Future research would benefit from oversampling healthcare professionals or examining more homogeneous subgroups. More generally, our professional sample comprised a wide range of occupations. While this heterogeneity enhances generalizability across work contexts, it may also have diluted effects specific to particular professions. Importantly, the aim of the present study was not to compare individual occupations, but to examine whether facet-level associations between playfulness, stress, and satisfaction generalize across broad academic and occupational contexts. Future studies could build on this work by focusing on more homogeneous occupational groups or by explicitly testing whether the relevance of specific playfulness facets depends on contextual features such as task structure, autonomy, or emotional labor.

Finally, we found moderate model fit, which indicates that replication with refined or context-specific playfulness measures is desirable.

## 5. Conclusions

Overall, the lighthearted and other-directed facets of playfulness may represent low-threshold personal resources that support mental health and satisfaction in both academic and occupational settings. These findings extend positive psychology perspectives in healthcare and education by emphasizing everyday playful mindsets rather than relying solely on structured leisure activities or clinical interventions.

From an applied perspective, lighthearted playfulness may be fostered through brief (self-directed) exercises that encourage playful reframing of everyday demands, for example by fostering lighthearted and socially playful attitudes to support more positive interpretations of challenges and strengthen interpersonal connections. At the same time, the associations we found were small and facet-specific, which indicates that playfulness is only one of several factors potentially contributing to well-being. Accordingly, well-being should not be viewed solely as a function of individual dispositions. Organizational factors such as workload, staffing, and social climate remain central in shaping mental health, especially in healthcare contexts [66]. Promoting well-being in demanding environments like healthcare therefore requires a dual focus approach that combines the cultivation of personal strengths with improvements to structural and social working conditions.

## Figures and Tables

**Table 1:** Sample Description.

	Students	Professionals
Sample size	635	499
Age in years	*M* = 23.06 *SD* = 3.59	*M* = 37.00 *SD* = 13.96
Education ^a^		
9–10 years of school	—	45 (9.0%)
12–13 years of school	496 (78.1%)	157 (31.5%)
Completed vocational training	24 (3.8%)	95 (19.0%)
Undergraduate university degree	103 (16.2%)	46 (9.2%)
Postgraduate university degree	10 (1.6%)	137 (27.5%)
Doctorate	—	13 (2.6%)
Other	2 (0.3%)	6 (1.2%)
Working	No: 35.9% Yes: 64.1%	20–24 h: 17.2% 25–34 h: 21.8% 35–40 h: 34.3% Over 40 h: 26.7% (*M* = 35.71, *SD* = 2.94)
Study Area ^b^/Profession	53.5% Psychology 21.3% Psychology related 25.2% Other fields	30.7% Business, finance 19.4% Education, social services 16.5% Health sciences 10.3% Agriculture, production 9.7% Culture, leisure 7.4% Technology, engineering 6.0% Public service, safety

Notes: ^a^ Undergraduate = completed bachelor’s degree; postgraduate = completed master’s degree, diploma, state examination. ^b^ Psychology = pure psychology; psychology related = programs that include psychology or are closely related (e.g., health psychology, organizational psychology, or psychology as a minor alongside majors like sociology or media studies); other fields = studies in other disciplines (e.g., physics, medicine).

**Table 2:** Descriptive Statistics and Correlations for all Study Variables.

	*M*	*SD*	ω/α	1	2	3	4	5	6	7	8
Playfulness											
1 Other-directed	4.89	0.91	0.72								
2 Lighthearted	3.86	0.99	0.76	0.30 ***							
3 Intellectual	4.02	0.79	0.62	0.47 ***	0.40 ***						
4 Whimsical	3.90	1.01	0.80	0.30 ***	0.38 ***	0.45 ***					
5 Stress	3.16	0.86	0.81	−0.01	−0.19 ***	−0.06	0.03				
6 Job/academic satisfaction	4.54	0.99	0.84	0.03	−0.01	0.01	−0.07 *	−0.29 ***			
7 Growth satisfaction	4.48	1.05	0.80	0.08 **	−0.03	0.05	0.00	−0.14 ***	0.70 ***		
8 Age	29.19	11.86	—	−0.27 ***	0.06 *	0.04	0.02	−0.02	0.11 ***	0.00	
9 Gender	—	—	—	0.19 **	0.53 ***	0.17 *	0.35 ***	−0.32 ***	0.05	0.03	0.26 ***

Notes: *N* = 1134. Cronbach’s α for the satisfaction scales as they had only two items, McDonalds’ ω for the other variables. Gender is reported as Cohen’s *d* from independent-samples *t*-tests. Positive/negative *d* values = higher scores for men/women. Boldface indicates statistically significant coefficients. * *p* < 0.05, ** *p* < 0.01, *** *p* < 0.001. Two-tailed.

**Table 3:** Measurement Invariance Across Students and Professionals, and Across Those With and Without Healthcare Affiliation.

	CFI	RMSEA	SRMR	*df*
Students vs. Professionals				
Configural	0.886	0.064	0.061	1146
Metric	0.889	0.062	0.062	1175
Scalar	0.878	0.061	0.063	1336
Healthcare Affiliation				
Configural	0.810	0.059	0.066	1146
Metric	0.810	0.058	0.067	1175
Scalar	0.805	0.058	0.068	1204

Notes: *n* = 635 students and 499 professionals. *n* = 154 with healthcare affiliation and 980 without healthcare affiliation.

**Table 4:** Multigroup SEM (Latent Variables) Testing Associations Between OLIW Facets and Indicators of Stress and Satisfaction While Controlling for Age and Gender.

	Stress				Job/Academic Satisfaction				Growth Satisfaction			
	Students		Professionals		Students		Professionals		Students		Professionals	
	β [95% CI]	*SE*	β [95% CI]	*SE*	β [95% CI]	*SE*	β [95% CI]	*SE*	β [95% CI]	*SE*	β [95% CI]	*SE*
Other-directed	0.13 [−0.04; 0.30]	0.09	−0.02 [−0.24; 0.21]	0.11	0.00 [−0.19; 0.18]	0.09	0.36 ** [0.13; 0.59]	0.12	0.05 [−0.13; 0.23]	0.09	0.32 ** [0.09; 0.56]	0.12
Lighthearted	−0.40 *** [−0.52; −0.28]	0.06	−0.28 *** [−0.43; −0.13]	0.08	−0.08 [−0.21; 0.06]	0.07	0.15 [0.00; 0.31]	0.08	−0.05 [−0.19; 0.09]	0.07	0.03 [−0.15; 0.20]	0.09
Intellectual	−0.01 [−0.23; 0.21]	0.11	0.07 [−0.18; 0.31]	0.13	0.11 [−0.14; 0.36]	0.13	−0.23 [−0.49; 0.03]	0.13	−0.04 [−0.28; 0.21]	0.12	−0.12 [−0.38; 0.15]	0.13
Whimsical	0.15 * [0.01; 0.29]	0.07	0.15 [−0.02; 0.33]	0.09	−0.12 [−0.28; 0.05]	0.08	−0.17 [−0.35; 0.00]	0.09	0.02 [−0.14; 0.17]	0.08	−0.06 [−0.24; 0.12]	0.09

Notes: *N* = 1134. *n*_Students_ = 635 and *n*_Professionals_ = 499. Reported coefficients are standardized regression coefficients (β) with 95% confidence intervals. * *p* < 0.05, ** *p* < 0.01, *** *p* < 0.001. Boldface indicates statistically significant coefficients.

## Data Availability

The original data presented in the study are openly available in OSF at https://osf.io/fa2zb (accessed on 22 December 2025).
